# Paliperidone Inducing Concomitantly Syndrome of Inappropriate Antidiuretic Hormone, Neuroleptic Malignant Syndrome, and Rhabdomyolysis

**DOI:** 10.1155/2016/2587963

**Published:** 2016-09-18

**Authors:** Jaspinder Kaur, Dileep Kumar, Mostafa Alfishawy, Ricardo Lopez, Issac Sachmechi

**Affiliations:** Department of Internal Medicine, Icahn School of Medicine at Mount Sinai, Queens Hospital Center, Jamaica, NY 11432, USA

## Abstract

Paliperidone, an active metabolite of risperidone, is a new atypical antipsychotic agent. Syndrome of inappropriate antidiuretic hormone (SIADH), neuroleptic malignant syndrome (NMS), and rhabdomyolysis are the uncommon side effects of psychotropic drugs. We report a case of 35-year-old male with schizoaffective disorder who was admitted for acute-on-chronic exacerbation of his psychotic disorder for which intramuscular paliperidone 234 mg injection was given. Two days later, the patient developed hyponatremic seizures secondary to SIADH which was treated with hypertonic saline. On the third day, he developed high grade fever and severe muscle rigidity with raised creatine phosphokinase (CPK) and liver enzymes levels. He was treated with dantrolene 100 mg, bromocriptine 2.5 mg, and lorazepam 2 mg. Our patient required management of the three rare conditions following treatment with paliperidone. This case highlights the need for health care providers to be aware of the rare, potentially life threatening but preventable hyponatremia, NMS, and rhabdomyolysis as a possible adverse effect of paliperidone.

## 1. Introduction

Paliperidone, an active metabolite of risperidone, is a new second-generation neuroleptic recently introduced to market as an antagonist of brain dopamine D_2_ and serotonin 5-hydroxytryptamine (5-HT_2A_) receptors. Food and Drug Administration (FDA) has approved it for the treatment of schizophrenia and schizoaffective disorders, whereas off-label uses include management of psychosis and bipolar disorder [1]. The safety of paliperidone was evaluated in six-week clinical trials performed prior to FDA approval. In these placebo-controlled double-blind trials, the most common adverse reactions were extrapyramidal symptoms (3–10%), tachycardia (9–22%), hyperkinesia (3–11%), hypertonia (1–6%), and drowsiness (4–13%). Other observed side effects were cardiac disorders (various cardiac rhythm disturbances, prolonged QTc interval, and orthostatic hypotension), gastrointestinal disorders, anxiety, sleep disorder, and headache [2].

We report a case of patient with schizoaffective disorder on paliperidone showing three concomitant different clinical adverse effects, “neuroleptic malignant syndrome (NMS), syndrome of inappropriate antidiuretic hormone (SIADH), and rhabdomyolysis.”

## 2. Case Report

A 35-year-old male with a history of schizoaffective disorder for six years, treated with oral paliperidone, was brought by emergency medical services for irrational behavior, paranoia, and aggression in the setting of noncompliance with psychiatric treatment for three months. He was hospitalized for acute-on-chronic exacerbation of his psychotic disorder for which intramuscular paliperidone 234 mg injection was given. He denied any other concomitant drug ingestion or alcohol abuse; however, he had a history of previous hospitalization for exacerbation of his psychosis due to his nonadherence towards his treatment.

Two days after admission, he developed an episode of generalized tonic clonic seizures lasting for less than 5 minutes and resolved spontaneously. The patient was transferred to ICU for hyponatremic seizures; pertinent laboratory values included serum sodium 108 mg/dL (136–146), serum osmolality 242 mosm/L (275–295), urine osmolality 438 mosm/L (50–1200), and anion gap metabolic acidosis pH 7.32/PCO_2_ 30/PO_2_ 78/AG 16. Other labs including vitamin B12 (442; 223–1132 pg/mL); folate (8.3; 5.9–24.8 ng/mL); and rapid plasma regain (RPR) were negative. His electroencephalogram (EEG) showed very slow background consistent with diffuse cerebral dysfunction possibly secondary to medication effect. Brain imaging showed no acute intracranial hemorrhage, mass defect, or definite lobar infarction. A diagnosis of euvolemic hyponatremia and hypo osmolality secondary to SIADH was made and he was treated with 3% hypertonic saline 15 gm/500 mL at 50 cc/hr.

On the third hospital day, he developed high grade fever of 102.2 F and severe muscle rigidity. Physical exam revealed his upper limbs were severely contracted. His laboratory data revealed elevated creatine phosphokinase (CPK) 34,548 IU/L (26–189); raised liver enzymes; lactate 6.3 (0.5–2.2); serum cortisol 77.03 *μ*g/dL (0.7–22.4); free T4 1.00 ng/dL (0.58–1.64); serum magnesium 2.08 mg/dL (1.7–2.7); and serum phosphorus 3.5 mg/dL (2.5–4.9). The patient was intubated for airway protection. Intravenous hydration with sodium chloride 0.9% and sodium bicarbonate 75 cc/hour was given to prevent acute kidney injury, and gradual improvement of his sodium level was noticed. A diagnosis of paliperidone induced SIADH, NMS, and rhabdomyolysis was made. He was treated with dantrolene 100 mg intravenously (IV) two times a day (BID) for two days; bromocriptine 2.5 mg orally (PO) via nasogastric tube three times a day (TID) for the first five days and then once daily (QD) for the next five days; and lorazepam 2 mg IV as needed (PRN). After two weeks of treatment with this regimen, all his labs normalized (Table 1) and he was transferred to psychiatric unit.

## 3. Discussion

Long acting injectable (LAI), paliperidone palmitate, is efficacious for the acute and maintenance treatment of schizoaffective disorder and is reasonably well tolerated as reported in the present case. It offers a guaranteed delivery system, does not require supplementation with oral antipsychotics, can be administered once monthly, does not require an additional precautionary observation period after the injection, and thus enhances adherence. However, the high acquisition cost of LAI will likely be an important obstacle to its routine use [3].

Many antipsychotic drugs have been reported to cause SIADH as reported in the current case. Various mechanisms though not yet clear have been suggested, including long-term D2 receptor supersensitivity resulting in ADH release [4], antipsychotic induced hypotension leading to ADH release through baroreceptor reflex, and serotonin mediated effects on central 5-HT2 and 5-HT1c receptors stimulating ADH secretion [5].

NMS is described as an idiosyncratic drug reaction that results in dysregulation of multiple neurochemical and neuroendocrine systems potentially culminating in an end-stage hypermetabolic syndrome. Dehydration, rapid rate of neuroleptic loading, depot neuroleptics, age extremes, prolonged restraints use, use of other medications with neuroleptics (especially lithium), poorly controlled neuroleptic-induced extrapyramidal symptoms (EPS), treatment resistant EPS, withdrawal of antiparkinsonian medications, affective disorder, alcoholism, organic brain syndrome or previous brain injury, extrapyramidal disorder, iron deficiency, and catatonia are the most common risk factors for NMS [6]. In our case, history of schizoaffective disorder, use of depot neuroleptic, and catatonia might have predisposed the patient to NMS.

The underlying pathophysiology of NMS is complex and is still debated among experts. Markedly sudden reduction in central dopaminergic activity resulting from D2 receptor blockade within the nigrostriatal, hypothalamic, and mesolimbic/cortical pathways might explain the rigidity, hyperthermia, and altered mental status, respectively. Secondly, sympathoadrenal hyperactivity resulting from the removal of tonic inhibition and defect in calcium regulatory proteins within the sympathetic nervous system may further play a key role in its pathogenesis. Thirdly, release of calcium from the sarcoplasmic reticulum of muscle cells with antipsychotic usage may possibly lead to increased muscle contractility and rigidity, muscle breakdown, and hyperthermia [7]. Moreover, it was recently hypothesized that long-term treatment with second-generation antipsychotics might cause imbalances in serotonergic neurotransmission, leading to sensitization towards them, and an excess of central serotonin could determine a “relative hypodopaminergic state” which might increase the risk of developing NMS [8]. However, Martino et al. reported that NMS induced by second-generation antipsychotics is characterized by lower incidence, lower clinical severity, and less frequent lethal outcome than NMS induced by first generation antipsychotics [8]; contrarily, our patient has a typical presentation with mental status alteration, rigidity, diaphoresis, hyperpyrexia, tremor, and other EPS but with a favorable evolution and complete recovery.

In psychiatric patients, rhabdomyolysis usually occurs in association with the intramuscular route of administration, mechanical restraints, and acute dystonia, or in the context of an episode of psychomotor agitation due to specific muscle dysfunction. Rhabdomyolysis induced by antipsychotics is usually associated with NMS, although it may occur alone. The period between the initiation of antipsychotic treatment and the onset of rhabdomyolysis may differ considerably from one case to another which can range between 2 and 5 days and one and a half and two years after starting antipsychotic treatment [9]. Moreover, both the hyponatremia and quick supplementation of sodium chloride can also cause rhabdomyolysis. The exact etiology of rhabdomyolysis secondary to hyponatremia is unclear, but several mechanisms have been proposed. First, lower osmolality of the extracellular fluid in acute hyponatremia causes cell swelling, and lowered transmembrane potential hours after the extrusion of intracellular potassium causes the release of CPK and myoglobin [10]. Secondly, the decreased extracellular sodium may disturb the Na-Ca exchange pumps which cause increased intracellular calcium and cell death by releasing proteases and lipase [11]. In our patient, rhabdomyolysis may be secondary to intramuscular antipsychotic use, NMS, and hyponatremia.

## 4. Conclusion

To the best of our knowledge, this is the first case which reports the occurrence of both NMS and SIADH in the same patient treated with paliperidone which further led to rhabdomyolysis. This states that second-generation antipsychotics have potential to cause SIADH, NMS, and rhabdomyolysis and thus highlights the need for health care providers to be aware of these rare, potentially life threatening but preventable conditions. Further studies to investigate the etiology of antipsychotics inducing SIADH and NMS are necessary as these drug induced conditions can be associated with significant morbidity and mortality. Patients treated with paliperidone warrant close clinical monitoring as well as monitoring of serum sodium and CPK.

## Figures and Tables

**Table 1:** Timeline changes in creatine phosphokinase (CPK) and sodium levels.

Timeline	CPK (26–189 IU/L)^*∗*^	Sodium (136–146 mg/dL)^*∗*^	Temperature	Leucocytes count (4.5–11.0K/*μ*L)^*∗*^	AST (10–40 IU/L)^*∗*^/ALT (7–50 IU/L)^*∗*^
Day 1	999	113	Afebrile	15.7	38/31
Day 3	31173	108 114	102.2 F	13.0	289/67
Day 4	34548	131	101.0 F	13.0	226/86
Week 1	5218	137	100.5 F	10.9	165/96
Week 2	288	135	Afebrile	8.1	63/93

^*∗*^Values in the brackets correspond to normal range.
